# Impact of 2 days of staging at 2500–4300 m on sleep quality and quantity following subsequent exposure to 4300 m

**DOI:** 10.14814/phy2.15063

**Published:** 2021-10-29

**Authors:** Janet E. Staab, Stephen R. Muza, Charles S. Fulco, Sean P. Andrew, Beth A. Beidleman

**Affiliations:** ^1^ Military Performance Division U.S. Army Research Institute of Environmental Medicine Natick Massachusetts USA; ^2^ Strategic Scientific Management Office U.S. Army Research Institute of Environmental Medicine Natick Massachusetts USA; ^3^ Thermal and Mountain Medicine Division U.S. Army Research Institute of Environmental Medicine Natick Massachusetts USA; ^4^ Biophysics and Biomedical Modeling Division U.S. Army Research Institute of Environmental Medicine Natick Massachusetts USA

**Keywords:** acclimatization, altitude, hypobaric hypoxia, sleep

## Abstract

The impact of 2 days of staging at 2500–4300 m on sleep quality and quantity following subsequent exposure to 4300 m was determined. Forty‐eight unacclimatized men and women were randomly assigned to stage for 2 days at one of four altitudes (2500, 3000, 3500, or 4300 m) prior to assessment on the summit of Pikes Peak (4300 m) for 2 days. Volunteers slept for one night at sea level (SL), two nights at respective staging altitudes, and two nights at Pikes Peak. Each wore a pulse oximeter to measure sleep arterial oxygen saturation (sSpO_2_, %) and number of desaturations (DeSHr, events/hr) and a wrist motion detector to estimate sleep awakenings (Awak, awakes/hr) and sleep efficiency (Eff, %). Acute mountain sickness (AMS) was assessed using the Environmental Symptoms Questionnaire and daytime SpO_2_ was assessed after AMS measurements. The mean of all variables for both staging days (STG) and Pikes Peak days (PP) was calculated. The sSpO_2_ and daytime SpO_2_ decreased (*p* < 0.05) from SL during STG in all groups in a dose‐dependent manner. During STG, DeSHr were higher (*p* < 0.05), Eff was lower (*p* < 0.05), and AMS symptoms were higher (*p* < 0.05) in the 3500 and 4300 m groups compared to the 2500 and 3000 m groups while Awak did not differ (*p* > 0.05) between groups. At PP, the sSpO_2_, DeSHr, Awak, and Eff were similar among all groups but the 2500 m group had greater AMS symptoms (*p* < 0.05) than the other groups. Two days of staging at 2500–4300 m induced a similar degree of sleep acclimatization during subsequent ascent to 4300 m but the 2500 m group was not protected against AMS at 4300 m.

## 1 INTRODUCTION

Sleep disturbances are common in newcomers following rapid ascent to high altitudes and are likely driven in part by an exaggerated peripheral chemoreceptor response which can lead to periodic breathing, frequent arousals, and a feeling of suffocation upon awakening (Ainslie et al., 2013; Bloch et al., 2015). The higher the altitude, the greater the disturbance in sleep (Ainslie et al., 2013; Pramsohler et al., 2019). Poor sleep is a major impediment when performing demanding physical and cognitive tasks at high altitude as it induces daytime drowsiness and fatigue (Aquino et al., 2012; Beaumont et al., 2007; Bian et al., 2019; Issa et al., 2016).

Recent reports document that men demonstrate greater sleep disturbances than women at high altitude (Caravita et al., 2015; Lombardi et al., 2013; Pramsohler et al., 2019). It is well known that sex hormones affect the respiratory centers both directly and indirectly (Behan & Wenninger, 2008). Progesterone stimulates ventilation (Behan & Wenninger, 2008; Gargaglioni et al., 2019) while estrogen increases cerebral blood flow (Krause et al., 2006) and one study documented a tight correlation between increased cerebral blood flow and decreased periodic breathing at high altitude (Ainslie et al., 2007). Both of these hormonal influences may lead to better sleep in women at altitude but further research is needed.

Physical activity has been reported to both improve (Aquino‐Lemos et al., 2016) and hinder (Tellez et al., 2016) sleep at altitude. Exercise typically exacerbates arterial hypoxemia (Fulco et al., 1988). For instance at 4300 m, it is not uncommon to see a 5%–10% decrease in pulse arterial oxygen saturation (SpO_2_) during moderate to intense exercise when compared to rest (Banchero et al., 1966). Whether this drop in SpO_2_ during physical activity carries over into sleep is unknown and research on the overall impact of physical activity on sleep disturbances at altitude is scant.

Whether sleep improves with altitude acclimatization is also controversial. Some have reported improvements in the quality and quantity of sleep with acclimatization (Fulco et al., 2011; Nussbaumer‐Ochsner et al., 2012; Reite et al., 1975) while others have reported little improvement (Latshang et al., 2013; Orr et al., 2018; Tseng et al., 2015). Differing results may depend on the severity of the altitude exposure with higher altitudes inducing a greater acclimatization response. Given that altitude acclimatization encompasses a series of integrated physiologic responses (e.g., increase in ventilation, decrease in plasma volume, and increase in sympathetic activity) that mitigate the reduction in oxygen delivery to the tissues (Young et al., 2002), it seems intuitive that acclimatization would result in improved sleep at altitude. Discrepant results between studies, however, indicate the need for further study.

“Staging” or residing for several days at an intermediate or moderate altitude prior to ascending to a higher elevation is a universally accepted acclimatization strategy (Beidleman et al., 2009; Fulco et al., 2009; Stamper et al., 1980). Previous research reported functional improvements in acute mountain sickness (AMS) symptomatology but not time trial performance during subsequent exposure to 4300 m following 2 days of staging at 2500–3500 m (Beidleman et al., 2018; Kenefick et al., 2019). However, the impact of staging at various altitudes on sleep quality and quantity in men and women under sedentary and physically active conditions has not been examined in a systematic manner.

As part of a comprehensive study examining the effectiveness of 2 days of staging at true altitudes on AMS and exercise performance following subsequent ascent to 4300 m (Beidleman et al., 2018; Kenefick et al., 2019), the sleep, ventilatory, cardiovascular, and AMS response were measured for 2 days while staging at 2500, 3000, 3500, and 4300 m and measured again for 2 days following subsequent ascent to 4300 m. We hypothesized that (1) staging at the higher altitudes (3500 and 4300 m) would be more stressful on the body (greater AMS and sleep disturbances) than staging at the lower altitudes (2500 and 3000 m), (2) two days of staging at 2500–4300 m would induce sleep and physiologic acclimatization during a subsequent exposure to 4300 m altitude in a dose‐dependent fashion with higher staging altitudes inducing a greater acclimatization response, (3) men would experience greater sleep disturbances than women both while staging and during subsequent exposure to 4300 m in all groups, and (4) engaging in physical activity while staging would increase sleep disturbances in all groups.

## 2 METHODS

The study was approved by the Institutional Review Board at the US Army Research Institute of Environmental Medicine (USARIEM) and the Human Research Protection Office, US Army Medical Research and Materiel Command. All volunteers provided written and verbal acknowledgment of their informed consent and were made aware of their right to withdraw without prejudice at any time. Investigators adhered to the policies for protection of human subjects as prescribed in Department of Defense Instruction 3216.02 and the research was conducted in adherence with 32 Code of Federal Regulations Part 219 on the use of volunteers in research.

### 2.1 Study volunteers

Sixty‐five unacclimatized men and women were originally enrolled in this study and were randomly assigned into one of four independent groups (each divided into a sedentary (S) and active (A) subgroup) that either resided for 2 days at 2500 m (*n* = 12, 560 mmHg), 3000 m (*n* = 12, 526 mmHg), 3500 m (*n* = 12, 494 mmHg), or 4300 m (*n* = 12, 460 mmHg) prior to assessments on the 3^rd^ and 4^th^ day of the study at 4300 m. Four dropped out after SL testing. Thirteen did not have sleep measurements either while staging and/or during exposure to 4300 m. The remaining 48 men and women were analyzed. Fortunately, in the remaining volunteers, a similar number of men and women compromised each staging group. Those in the S group engaged in a total of 3 h of a combination of board/card games twice daily (i.e., morning and afternoon) while those in the A group participated in a ~90‐min trail hike twice daily conducted at approximately 5–6 metabolic equivalents while staging. During PP exposure, all volunteers participated in the same activities. All volunteers were healthy, well nourished, physically active, non‐smokers with hematologic and ferritin values in the normal range. Age range was limited to 18–39 year olds. None had been diagnosed with a sleep disorder. All were born at altitudes <1500 m and none had been living at altitudes >1000 m in the previous 3 months prior to the start of the study. Physical characteristics were determined at SL (756 mmHg) and are presented in Table 1 for the entire cohort as well as by altitude staging group. Our results are limited to the physical characteristics of our study population.

**TABLE 1:** Physical characteristics of unacclimatized lowlanders assigned to one of four altitude staging groups

	Entire Cohort	2500 m	3000 m	3500 m	4300 m
Volunteers	*N* = 48	*n* = 12	*n* = 12	*n* = 12	*n* = 12
Age (yrs)	23 ± 1	24 ± 2 [19–39]	23 ± 1 [18–31]	24 ± 1 [19–31]	22 ± 1 [18–30]
Height (cm)	173 ± 1	173 ± 3 [155–186]	175 ± 3 [159–186]	172 ± 3 [159–183]	173 ± 2 [159–187]
Weight (kg)	71 ± 2	71 ± 4 [51–86]	77 ± 4 [62–96]	70 ± 3 [50–89]	73 ± 3 [54–95]
VO_2peak_ (ml/min/kg)	50 ± 1	47 ± 1 [38–54]	50 ± 2 [39–67]	49 ± 2 [41–60]	52 ± 2 [38–66]
Active (A)	*N* = 25	*n* = 5	*n* = 7	*n* = 6	*n* = 7
Sedentary (S)	*N* = 23	*n* = 7	*n* = 5	*n* = 6	*n* = 5
Males	*N* = 33	*n* = 8	*n* = 8	*n* = 9	*n* = 8
Females	*N* = 15	*n* = 4	*n* = 4	*n* = 3	*n* = 4

Means ± Standard Error (SE); [Range], VO_2peak_, peak oxygen uptake.

### 2.2 Study design

**FIGURE 1:**
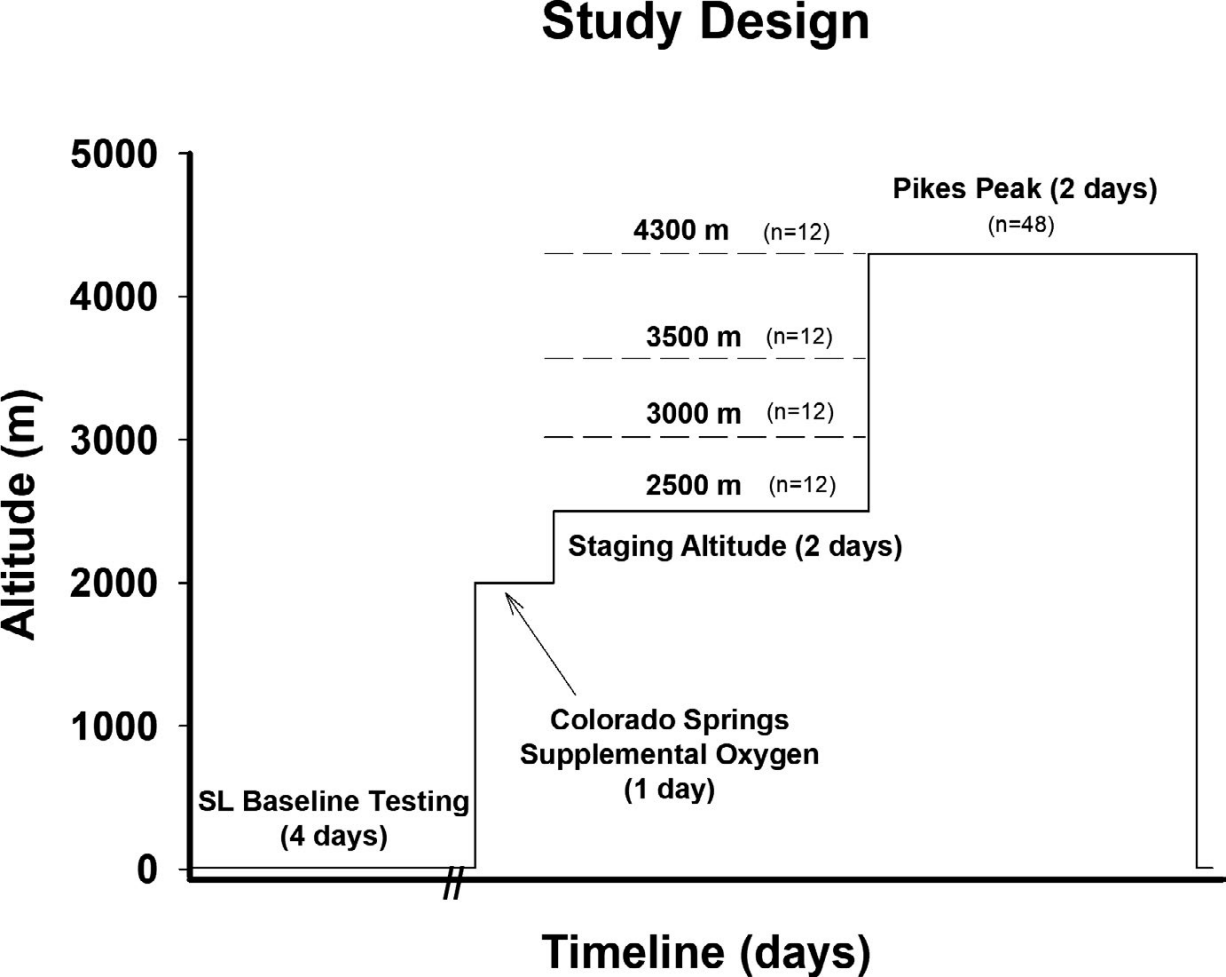
Study design. All volunteers completed four days of sea level (SL) baseline testing and then were flown to Colorado Springs where they slept for one night on supplemental oxygen to simulate SL conditions. The next day they were removed from supplemental oxygen and driven to one of four altitude sites (e.g., 2500 m, 3000 m, 3500 m or 4300 m) where they resided for the next two days and participated in sedentary and vigorous activities. On the third morning, the 2500 m, 3000 m and 3500 m groups were driven by car to the laboratory on the summit of Pikes Peak (4300 m). Sleep measurements were initiated around 10:00 pm on the first (STG1) and second night (STG2) of staging and first (PP1) and second (PP2) night at the Pikes Peak (PP) laboratory. Acute Mountain Sickness (AMS) measurements were initiated ~every 4 h (8 am, 12 pm, 4 pm and 8 pm) at STG1, STG2, PP1, and PP2

This study utilized a prospective, randomized design (Figure 1). Following 4 days of baseline SL testing, volunteers were flown in the morning from Boston, MA to Colorado Springs, CO (1885 m) where they spent the remainder of the day and night in an apartment breathing supplemental O_2_. The SpO_2_ (%) was continuously monitored via pulse oximetry in the apartment in Colorado Springs and mean values were maintained at SL values in the 2500 m (98 ± 2), 3000 m (97 ± 3), 3500 m (98 ± 2), and 4300 m groups (97 ± 2) from their time of arrival until their time of departure at 0500 h the next morning. After volunteers were removed from supplemental O_2_, they were driven to their respective altitude staging sites (e.g., 2500, 3000, 3500, or 4300 m) located within the Pike National Forest where they stayed for 2 days. Sleep measurements were initiated around 10:00 pm on the first (STG1) and second (STG2) night at the respective staging altitudes. AMS, SpO_2_, and HR measurements were taken ~every 4 h on STG1 and STG2 (8 am, 12 pm, 4 pm, and 8 pm) and the peak AMS symptom scores on each day were calculated. On the morning of the 3^rd^ day, the 2500, 3000, and 3500 m groups were driven for 1–3 h to the Pikes Peak laboratory (4300 m, 446 mmHg). All arrived between 7:30 and 10:00 am and sleep measurements were initiated around 10:00 pm on the first (PP1) and second (PP2) night. AMS, SpO_2_, and HR measurements were taken ~every 4 h on PP1 and PP2 (8 am, 12 pm, 4 pm, and 8 pm) and the peak AMS scores on each day were calculated The 4300 m group had already been residing at the Pikes Peak laboratory for the previous 2 days and the same AMS and sleep measurements were initiated on their 3^rd^ day of residence at the same time of day as the other staging groups.

### 2.3 Research procedures

#### 2.3.1 Peak oxygen uptake (VO_2peak_)

An incremental, progressive exercise bout to volitional exhaustion on a treadmill was used to assess VO_2peak_ during the USARIEM SL baseline phase. Measurements of O_2_ uptake were obtained using a metabolic cart (True Max 2400, Parvo Medics, Sandy, UT) using a previously described protocol (Kenefick et al., 2019).

#### 2.3.2 Sleep measures

An actigraph was used to differentiate sleep from wakefulness based on wrist movement (Ambulatory Monitoring, Inc.). Estimation of sleep by actigraphy has a high level of correlation with sleep estimation by polysomnography (Cole et al., 1992; Souza et al., 2003). Study subjects wore the actigraph on the wrist of their dominant hand and pressed an event button when they lay down to sleep and again on waking up in the morning. Recordings between the two events were analyzed for sleep awakenings (Awak, events/hour), total time in bed (min), total sleep time (min), and sleep efficiency (Eff, %). Eff was calculated as total sleep time divided by the total time in bed and multiplied by 100. Data were recorded using zero crossing mode (ZCM) in 1‐minute epochs. Automated analysis was performed using the Cole‐Kripke algorithm on the ZCM channel at a 1‐minute sample rate (Action 4 Version 1.13, Ambulatory Monitoring Inc.) (Cole et al., 1992; Souza et al., 2003).

A pulse oximeter worn on the non‐dominant hand was used to measure sleep pulse oxygen saturation (sSpO_2_) and sleep heart rate (sHR) (Nonin 3100 WristOx, Nonin Medical, Inc.). Study subjects were instructed to put on the pulse oximeter when they went to bed and removed the pulse oximeter the next morning when they got out of bed. Compatible software was used to determine sleep pulse oxygen saturation (sSpO_2_, %), sleep heart rate (sHR, beats/min), and relative desaturations (DeSHr, events/hr) defined as ≥4% drop in oxygen saturation for a minimum of 10 s recorded (nVISION Version 6.3j, Nonin Medical, Inc.). All volunteers wore the same actigraph and pulse oximeter throughout the study.

#### 2.3.3 Acute mountain sickness (AMS)

The severity and prevalence of AMS were determined from information gathered from the shortened version of the Environmental Symptoms Questionnaire (ESQ) (Beidleman et al., 2007; Sampson et al., 1983) after 4, 8, 12, 22, 28, 32, 36, and 46 h of residence at the staging altitudes and Pikes Peak. The cerebral factor score (AMS‐C) was calculated from the ESQ and if AMS‐C was ≥0.7, the subjects were considered sick with AMS. At the completion of each questionnaire, and with the volunteer still seated, SpO_2_ and HR were also measured (Model 9560, Nonin Medical, Plymouth, MN). The mean SpO_2_ and HR (mSpO_2_ and mHR) as well as the peak AMS severity and prevalence were calculated across the four measurement points on each of the staging days (STG1 and STG2) and at Pikes Peak (PP1 and PP2).

#### 2.3.4 Activity

Volunteers continuously wore a small ankle‐sized one‐directional accelerometer activity monitor (Actical, Philips Respironics, Murrysville, PA) throughout the study which has been shown to provide reliable and valid measurements of energy expenditure when compared with indirect calorimetry (Hager et al., 2015). The activity monitor was removed for daily showers and used to calculate total daily energy expenditure (kcal/d) for the S and A groups each day of the study.

#### 2.3.5 Diet

During the entire study, all volunteers were allowed to eat ad libitum. While residing at their SL residence, volunteers ate their typical meals at home. While residing at altitude, the volunteers ate Meal Ready‐to‐Eat rations supplemented with common foods such as vegetables, fruits, milk, and juices. Considerable efforts were made to sustain caloric balance and hydration by providing similar food (macronutrient composition) and water during data collection before and throughout their altitude exposures. Urine‐specific gravity was <1.002 each morning for all volunteers indicating that they were euhydrated throughout testing.

#### 2.3.6 Statistics

A 4 (staging group) by 2 (activity level or sex) by 3 (condition) repeated measures linear or generalized linear mixed model was used to analyze the data with group (2500, 3000, 3500, or 4300 m), activity level (S vs. A), or sex (men vs. women) as independent factors and condition (SL, STG, and PP) as the repeated measures factor using SAS PROC Mixed (SAS 9.3, Cary NC). Activity level and sex were evaluated in separate analyses due to limited women undergoing different physical activity conditions in each group. Data normality was tested using the Shapiro–Wilk test and if parameters failed to meet normality assumptions, the data were treated with appropriate nonparametric statistics. Pearson correlation coefficients were calculated for the relationship between AMS‐C and sleep parameters in each condition. Statistical power calculations indicated that a sample of 8–12 subjects per group would provide a > 80% chance of detecting a change in sleep quality and quantity based on previous research at the various altitudes examined in this study (Fulco et al., 2011; Jones et al., 2008; Latshang et al., 2013; Muhm et al., 2009; Pramsohler et al., 2019). Significant main effects and interactions were analyzed using a Neuman–Keuls post hoc test. Significance was set at *p* < 0.05. Data are presented as means ± standard error (SE).

## 3 RESULTS

There were no differences (*p* > 0.05) between groups in any of their physical characteristics (Table 1). The composition of the groups in terms of activity level (S vs. A) and sex (men vs. women) was not different. The total energy expenditure (kcal/d) was lower (*p* < 0.05) in the S versus A subgroups at 2500 m (3131 ± 196 vs. 4010 ± 210), 3000 m (3122 ± 226 vs. 3958 ± 202), 3500 m (2801 ± 226 vs. 3749 ± 220), and 4300 m (2893 ± 252 vs. 3365 ± 211). Activity level did not affect any of the outcome variables so data from both the S and A groups were combined for further analyses. There were no significant three‐way interactions between group, sex, and condition but significant two‐way interactions between group and condition (with both sexes combined) and sex and condition (with all groups combined) were examined in further detail.

### 3.1 Sleep measures by group and condition

**FIGURE 2:**
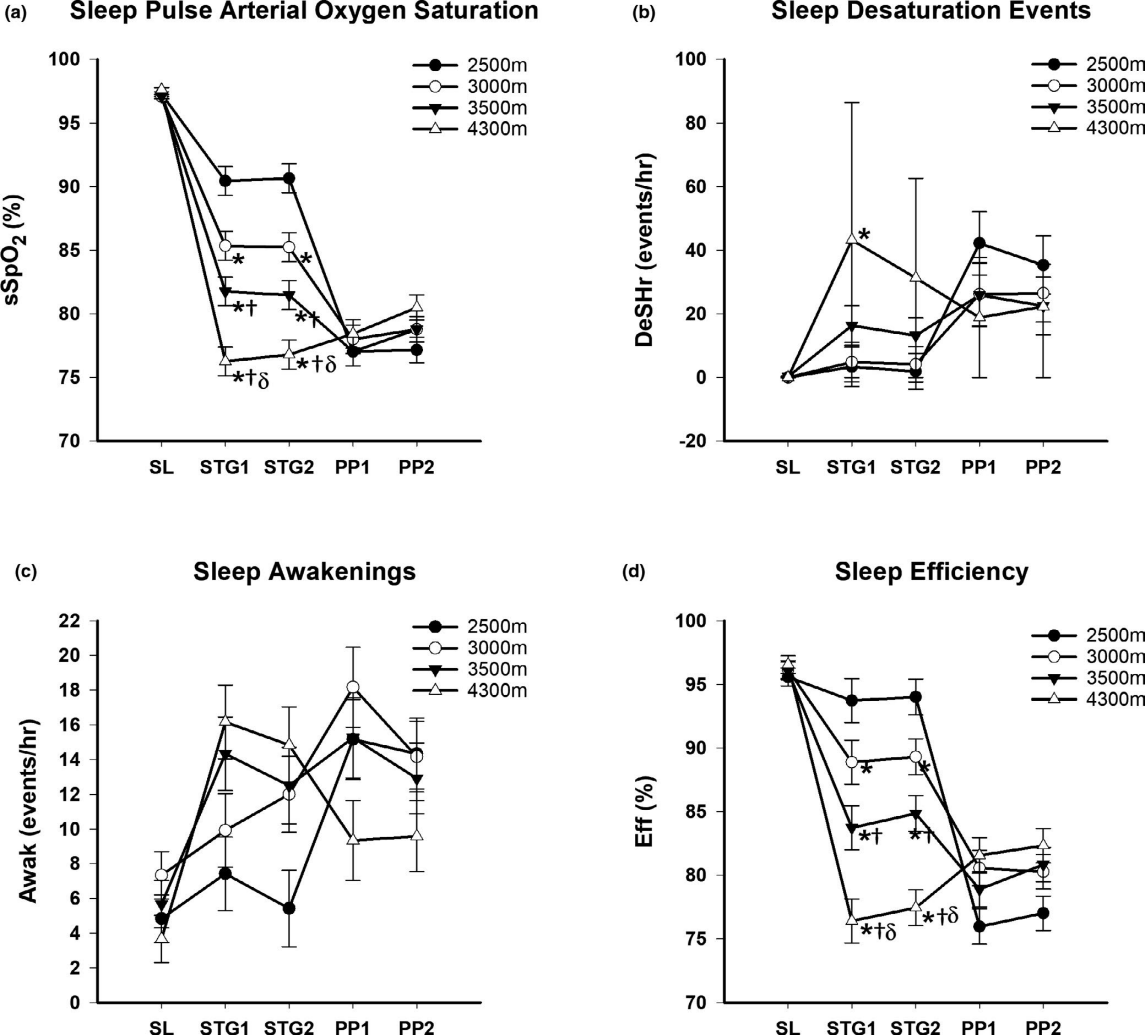
(a) Mean ± SE. Sleep pulse arterial oxygen saturation (sSpO2, %) at sea level (SL), Stage Day 1 (STG1), Stage Day 2 (STG2), Pikes Peak Day 1 (PP1) and Pikes Peak Day 2 (PP2), (b) Mean sleep desaturation (DeSHr, events/hr) at SL, STG1, STG2, PP1 and PP2, (c) Mean sleep awakenings (Awak, events/hr) at SL, STG1, STG2, PP1 and PP2 and (d) Mean total sleep efficiency (Eff, %) at SL, STG1, STG2, PP1 and PP2 in four independent groups staged for two days at 2500, 3000, 3500 or 4300 meters (m) prior to ascent and/or stay at 4300 m. *p < 0.05 from 2500 m. †*p* < 0.05 from 3000 m. δ*p* < 0.05 from 3500 m

Figure 2 presents the sleep sSpO_2_ (%), DeSHr (events/hr), Awak (events/hr), and Eff (%) in all four groups at SL, STG1, STG2, PP1, and PP2. The sSpO_2_ decreased (*p* < 0.05) from SL values during both STG1 and STG2 in an altitude‐dependent fashion with significant differences between all groups. The 4300 m group had a greater (*p* < 0.05) number of DeSHr at STG1 than the 2500 and 3000 m groups. The Eff decreased (*p* < 0.05) from SL during STG1 and STG2 in the 3500 and 4300 m groups but not in the 2500 and 3000 m groups. All groups had similar sSpO_2_, DeSHr, and Eff at PP1 and PP2. There was no difference in Awak at STG1, STG2, PP1, or PP2 between groups.

The sHR increased (*p* < 0.05) from SL at STG1 and STG2, respectively, in the 2500 m (63 ± 3; 62 ± 3), 3000 m (67 ± 3; 68 ± 3), 3500 m (71 ± 3; 71 ± 3), and 4300 m (75 ± 3; 73 ± 3) groups in a dose‐dependent fashion. The sHR was higher (*p* < 0.05) in the 4300 m group compared to the 2500 m group both at STG1 and STG2. Upon exposure to PP1 and PP2, respectively, there were no group differences in the sHR in the 2500 m (82 ± 3; 83 ± 3), 3000 m (78 ± 3; 76 ± 3), 3500 m (81 ± 3; 80 ± 3), and 4300 m (76 ± 3; 73 ± 3) groups.

### 3.2 *Sleep measures by sex and condition*:

**FIGURE 3:**
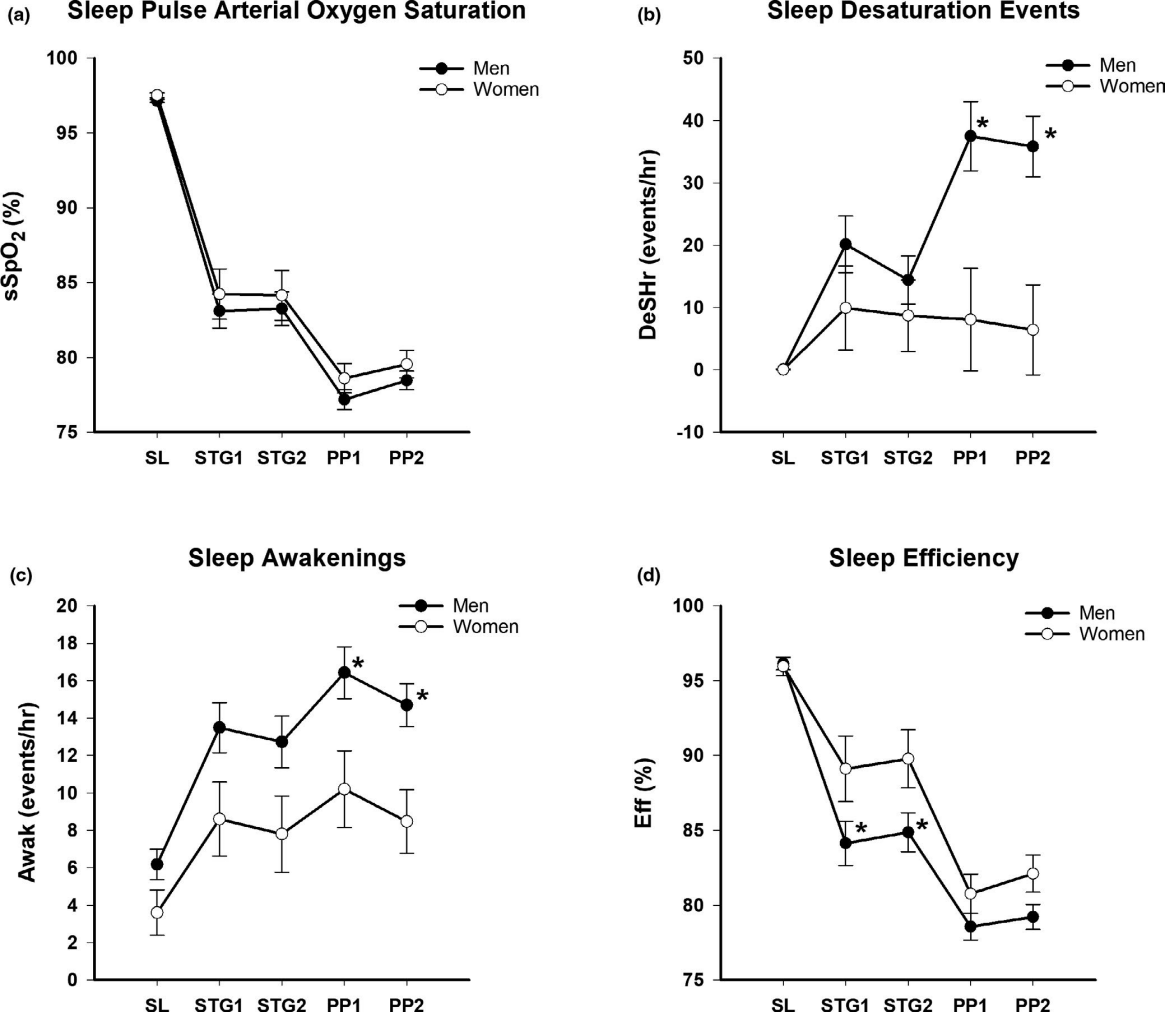
(a) Mean ± SE Sleep pulse arterial oxygen saturation (sSpO2, %) at sea level (SL), Stage Day 1 (STG1), Stage Day 2 (STG2), Pikes Peak Day 1 (PP1) and Pikes Peak Day 2 (PP2) in men and women, (b) Mean sleep desaturation (DeSHr, events/hr) at SL, STG1, STG2, PP1 and PP2 in men and women, (c) Mean sleep awakenings (Awak, events/hr) at SL, STG1, STG2, PP1 and PP2 in men and women and (d) Mean total sleep efficiency (Eff, %) at SL, STG1, STG2, PP1 and PP2 in men and women staged for two days at 2500, 3000, 3500 or 4300 meters (m) prior to ascent and/or stay at 4300 m. *p < 0.05 from females

Figure 3 presents the sSpO_2_ (%), DeSHr (events/hour), Awak (events/hour), and Eff (%) in men and women in each of the conditions. There were no differences in sSpO_2_ between men and women in any condition but the Eff at STG1 and STG2 was lower (*p* < 0.05) in men compared to women while DeSHr and Awak were higher (*p* < 0.05) in men versus women at PP1 and PP2.

### 3.3 *Acute mountain sickness measures by group and condition*:

**FIGURE 4:**
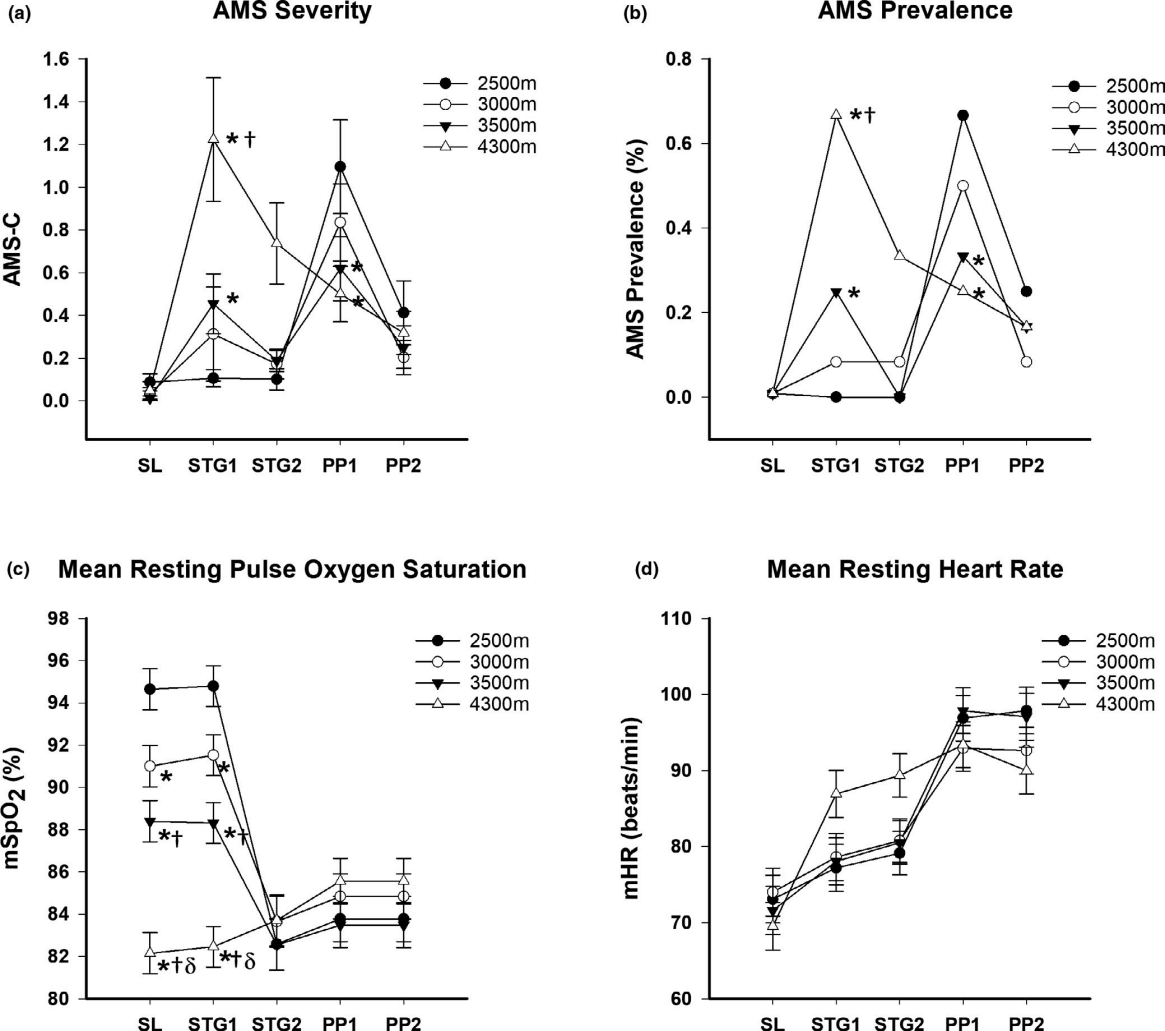
(a) Mean ± SE Acute Mountain Sickness cerebral factor score (AMS‐C) at sea level (SL), Stage Day 1 (STG1), Stage Day 2 (STG2), Pikes Peak Day 1 (PP1) and Pikes Peak Day 2 (PP2), (b) AMS prevalence (%) at SL, STG1, STG2, PP1 and PP2, (c) Mean resting pulse oxygen saturation (mSpO2, %) at SL, STG1, STG2, PP1 and PP2 and (d) Mean resting heart rate (mHR, beats/min) at SL, STG1, STG2, PP1 and PP2 in four independent groups staged for two days at 2500, 3000, 3500 or 4300 meters (m) prior to ascent and/or stay at 4300 m. *p < 0.05 from 2500 m. †*p* < 0.05 from 3000 m. δ*p* < 0.05 from 3500 m

Figure 4 presents the peak severity and prevalence of AMS as well as the mSpO_2_ and mHR in each of the conditions. There was no effect of sex on AMS so both sexes were combined. Peak AMS severity and prevalence increased (*p* < 0.05) from SL at STG1 in the 3500 and 4300 m groups. Upon exposure to PP1 and PP2, the 3000, 3500, and 4300 m groups exhibited similar peak AMS severity and prevalence but the 2500 m group had a higher peak severity and prevalence at PP1 compared to the 4300 m group. The mSpO_2_ decreased (*p* < 0.05) from SL values during STG1 and STG2 in an altitude‐dependent fashion with significant differences between groups. The mHR increased (*p* < 0.05) from SL values during STG1 and STG2 only in the 3500 and 4300 m groups. There were no group differences in mSpO_2_ or mHR at PP1 and PP2.

### 3.4 *Physiologic and sleep acclimatization*:

There were no differences in mHR and mSpO_2_ from STG1 to STG2 or PP1 to PP2 in any of the groups (Figure 4). Given the change in altitude in the lower three staging groups, physiologic acclimatization from day 1 to day 4 was not assessed in these groups. The mHR decreased (*p* < 0.05) and mSpO_2_ increased (*p* < 0.05), however, from day 1 to day 4 in the 4300 m group. In terms of sleep acclimatization (Figure 2), none of the lower staging groups improved any measures of sleep quality and quantity from STG1 to STG2 or PP1 to PP2 but the 4300 m group demonstrated an increased sSpO_2_ and Eff and decreased sHR from day 1 to day 4 at Pikes Peak.

### 3.5 *Relationships between sleep and AMS*:

In all groups, there was a significant correlation between mean resting (mSpO_2_) and sleep SpO_2_ (sSpO_2_) at STG1 (*r* = 0.92; *p* = 0.001), STG2 (*r* = 0.92; *p* = 0.001), PP1 (*r* = 0.79; *p* = 0.002), and PP2 (*r* = 0.79; *p* = 0.002). The sSpO_2_ was ~5%–6% lower than mSpO_2_ in all groups and conditions. In all groups, there was also a significant correlation between mHR and sHR at STG1 (*r* = 0.81; *p* = 0.001), STG2 (*r* = 0.79; *p* = 0.001), PP1 (*r* = 0.75; *p* = 0.001), and PP2 (*r* = 0.74; *p* = 0.001). The sHR was ~13–16 beats/min lower than mHR in all groups and conditions.

**FIGURE 5:**
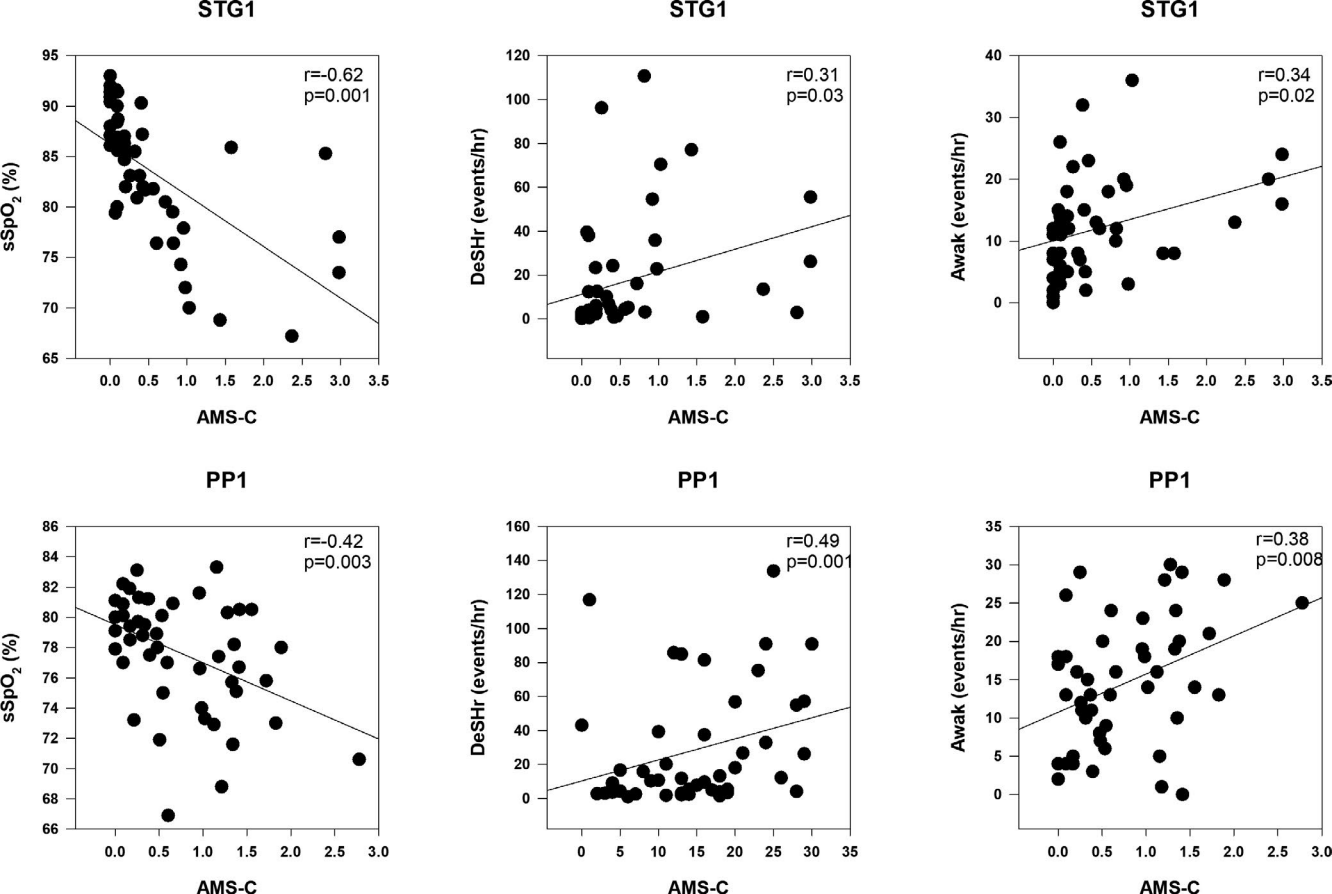
Correlations between Acute Mountain Sickness cerebral factor score (AMS‐C) and sleep pulse arterial oxygen saturation (sSpO2, %), desaturation events (DeSHr, events/hr) and sleep awakenings (Awak, events/hr) on the first day of staging (STG1) and the first day at Pikes Peak (PP1)

Figure 5 demonstrates that sleep sSpO_2_, DeSHr, and Awak were significantly correlated (*p* < 0.05) with AMS‐C severity scores at STG1 and PP1 when all groups were combined. Sleep quality and quantity measures were not significantly correlated with AMS‐C at STG2 and PP2. The Eff was also significantly correlated with AMS severity at STG1 (−0.49, *p* = 0.001) but not with PP2 while sHR was correlated with AMS severity at STG1 (*r* = 0.33, *p* = 0.02), STG2 (*r* = 0.31, *p* = 0.01), and PP1 (*r* = 0.30, *p* = 0.04).

## 4 DISCUSSION

These findings demonstrate that 2 days of staging at 2500–4300 m induced a similar degree of sleep and physiologic acclimatization during subsequent exposure to 4300 m. This finding was contrary to our hypothesis that higher staging altitudes would result in greater acclimatization following subsequent ascent to 4300 m. In agreement with our hypothesis, however, the 3500 and 4300 m groups experienced greater sleep disturbances and AMS while staging than the 2500 and 3000 m groups. In addition, the 2500 m group was not protected against AMS following subsequent ascent to 4300. Therefore, 3000 m may be the optimal 2‐day staging altitude to induce acclimatization and protect against AMS following subsequent ascent to 4300 m. Men experienced greater oxygen desaturation and awakenings during sleep than the females when all groups were combined and engaging in physical activity did not affect any of the sleep parameters. Sleep acclimatization did not occur over the 4 days of exposure in the lower altitude staging groups (2500, 3000, and 3500 m) but did occur in the 4300 m group. On the other hand, acclimatization, as measured by a reduction in AMS symptomatology, occurred over 4 days of exposure in all groups.

To our knowledge, this is the first study that has examined the impact of 2 days of staging at various moderate altitudes (2500–3500 m) on sleep quality and quantity following subsequent ascent to a high altitude (4300 m). Although several studies have examined sleep during high‐altitude mountaineering (Bloch et al., 2009; Ortiz‐Naretto et al., 2018; Tannheimer et al., 2017), the ascent profiles, medication use, physical activity during ascent, and final altitude reached differ dramatically among studies. These differences in study design make quantitative comparisons on the effectiveness of different staging strategies difficult at best. By manipulating the dose of acclimatization, via exposure to differing levels of hypoxia, while controlling for the total time of exposure, sex, and physical activity levels, the impact of staging on sleep during subsequent ascent to a higher altitude was examined in a systematic manner.

*Staging Results*: Several studies as well as review articles have reported an increase in sleep disturbances with increasing severity of altitude exposure (Ainslie et al., 2013; Bloch et al., 2015). This research also clearly delineates a deterioration in nocturnal sleep saturation and efficiency above 3000 m with no sleep disturbances detected at or below 3000 m. Most likely, this finding is due to the fact that altitudes greater than 3000 m lie on the steep portion of the oxygen dissociation curve. Others have also reported no differences in sleep quality and quantity at 3000 m and below (Latshang et al., 2013; Mizuno et al., 1993). The staging altitudes utilized in this study were effective in inducing different levels of hypoxemia as evidenced by an altitude‐dependent decrease in nocturnal SpO_2_ between groups. Our nocturnal SpO_2_ and HR values were similar to values reported in the literature by other investigative teams at similar altitudes. For instance, nocturnal SpO_2_ has been reported to range from 86% to 90% at ~2500 m (Latshang et al., 2013; Muhm et al., 2009), 83% to 85% at ~3000 m (Mizuno et al., 1993; Tannheimer et al., 2017), 81% to 83% at ~3500 m (Beaumont et al., 2007; Heinzer et al., 2016; Lombardi et al., 2013), and 71% to 76% at ~4300 m (Fulco et al., 2011; Jones et al., 2008; Reite et al., 1975). The ~10 beat/min increase in sHR observed in the 4300 m group in this study is consistent with the literature (Fulco et al., 2011; Jones et al., 2008; Reite et al., 1975). The lack of an increase in nocturnal HR in this study at the lower altitudes agrees with the results of some (Muhm et al., 2009) but not others (Kohler et al., 2008; Latshang et al., 2013). The discrepancy between studies may be related to the fact that the compensatory cardiovascular response to the initial hypoxic insult at the lower altitudes may be more subtle and less likely to demonstrate significance when small subject numbers are utilized.

Although wrist actigraphy and pulse oximetry were used to assess sleep quality and quantity, sleep measurements using these devices demonstrate a high correlation with polysomnography measurements (Latshang et al., 2016; Nussbaumer‐Ochsner et al., 2011). Studies that have employed polysomnography to study sleep at altitudes similar to the ones utilized in this study have reported an increase in desaturation events at ~3500 m (Beaumont et al., 2007; Fischer et al., 2004; Heinzer et al., 2016; Kohler et al., 2008) and above (Nussbaumer‐Ochsner et al., 2012; Orr et al., 2018) with no differences observed below this altitude (Latshang et al., 2013; Ortiz‐Naretto et al., 2018). In this study, sleep DeSHr were only increased in the 4300 m group during STG which differs from our hypothesis that both the 3500 and 4300 m groups would experience a greater number of these events. There is a high variability associated with this measurement; as well as differing definitions for what constitutes a desaturation event (>3% or >4%) (Ruehland et al., 2009). Depending on the definition utilized to analyze the data, results may vary with less desaturations expected when using the higher threshold (Heinzer et al., 2016). Given the ~threefold increase in DeSHr in the 3500 m group compared to the 2500 and 3000 m groups, the high variability and higher threshold may have precluded a statistically significant finding.

Sleep Awak did not change in any of the groups during STG in this study. These results are consistent with others that reported no change in sleep arousals or awakenings up to 4500 m (Ortiz‐Naretto et al., 2018; Rojc et al., 2014) but differs from others that reported increased awakenings above 3000 m (Beaumont et al., 2007; Tseng et al., 2015). The lack of consistency between studies in regards to sleep arousals is not well understood but may be related to the controversy over whether periodic breathing results in awakenings (Ainslie et al., 2013; Bloch et al., 2015). Sleep efficiency in this study decreased from SL values (96%) during STG1 at 2500 m (94%), 3000 m (89%), 3500 m (85%), and 4300 m (76%) in an altitude‐dose dependent fashion. There is a wide range of sleep efficiencies reported in the literature at SL (87%–97%) (Beaumont et al., 2007; Heinzer et al., 2016; Latshang et al., 2013; Muhm et al., 2009), ~2500 m (84%–94%) (Fulco et al., 2011; Muhm et al., 2009), ~3000 m (82%–92%) (Beaumont et al., 2007; Tseng et al., 2015), ~3500 m (81%–91%) (Heinzer et al., 2016; Kohler et al., 2008), and ~4300 m (71%–81%) (Beaumont et al., 2004; Fulco et al., 2011) and our values lie within these ranges. The large variation in sleep efficiency is most likely due to the large individual variability in sleep responses even when measured at SL which then translates to variability during altitude exposure. Overall, our results are remarkably consistent with polysomnography measurements taken at the same altitudes by different research teams which supports using these types of devices to assess the quality and quantity of sleep in the field.

Acute mountain sickness (AMS) has been well‐described and reviewed in the literature (Hackett & Roach, 2001). In general, symptoms include headache, gastrointestinal symptoms (nausea, anorexia, or vomiting), lassitude or fatigue, lightheadedness or dizziness, and insomnia or trouble sleeping (Hackett & Roach, 2001) although the updated Lake Louise Questionnaire (LLQ) eliminates the trouble sleeping question (Roach et al., 2018). The LLQ is probably the most widely utilized questionnaire to assess AMS due to its ease of use but the scoring is problematic. Some researchers use a cutoff value of ≥3 plus headache to define AMS (Roach et al., 1993) while others utilize ≥4 plus headache (Maggiorini et al., 1998), and yet others utilize ≥5 plus headache (Wagner et al., 2012). The ESQ has been used for over 37 years, the cutoff value for identifying AMS using the cerebral factor score has not changed over this time frame, and the questionnaire has a long history of evaluating the different factors associated with AMS separately (Sampson et al., 1983). The shortened electronic version of the ESQ was utilized in this study to assess AMS (Beidleman et al., 2007; Sampson et al., 1983) and has never included a sleep question in the calculation of AMS‐C which eliminates these issues.

Both AMS and sleep disturbances occur at altitude and most studies have reported a relationship between the two mechanistically separate issues at altitudes >2500 m due to the fact that hypoxia is the underlying cause of both problems (Nussbaumer‐Ochsner et al., 2012; Tseng et al., 2015). This study also found a significant relationship between AMS severity and all measures of sleep quality and quantity during STG1 but not STG2. AMS symptomatology improved on the 2^nd^ day of staging in the 3500 and 4300 m group while sleep disturbances remained unchanged which likely accounts for the lack of correlation between measures at STG2 and also suggests that AMS is not contributing to the sleep disturbances and vice versa.

The severity and prevalence of AMS during staging at 2500 m (0%), 3000 m (9%), 3500 m (33%), and 4000 m (67%) in this study differs slightly from our previously published results from the same study (Beidleman et al., 2018) due to analyzing a subset of the subjects (*n* = 48) that completed sleep assessments in all conditions. The prevalence of AMS observed in this study, however, aligns closely with others examining AMS at the same altitudes (Fulco et al., 2011; Maggiorini et al., 1998) as well as predictions from a quantitative model (Beidleman et al., 2013). Significant increases in both AMS severity and prevalence from SL did not occur until exposure to 3500 and 4300 m in this study. Resting mSpO_2_ and mHR, collected during AMS measurements while staging (Figure 4), also demonstrated that mHR did not increase until 3500 m and above. Together, the AMS and mHR results along with the sleep measures demonstrate that staging at 3500–4300 m is more stressful on the body than staging at the lower altitudes.

*Pikes Peak Results*: The overarching goal of this study was to examine the impact of 2 days of staging at four different altitudes (e.g., 2500, 3000, 3500, and 4300 m) on sleep quality and quantity following subsequent ascent to 4300 m. In terms of sleep parameters, all four altitudes were equally effective for inducing sleep acclimatization. The results were remarkably consistent with no group demonstrating a clear or distinct advantage over another group. When examining the resting physiologic responses (Figure 4), the resting responses were consistent and supportive of the sleep responses with each group achieving similar acclimatization. There was one exception to this pattern in that the 2500 m group experienced significant AMS during exposure to PP while the other staging groups were protected.

Neither AMS symptomatology nor sleep improved from PP1 to PP2 in any of the groups. However, both AMS and sleep improved over 4 days of acclimatization at 4300 m. All measures of sleep quality and quantity were significantly correlated with AMS severity measurements at PP1 but not PP2. Similar to staging, this lack of correlation is most likely due to the fact that AMS improved from PP1 to PP2 but sleep quality and quantity did not change. Others have reported that sleep acclimatization occurs at altitudes above 4000 m (Fulco et al., 2011; Nussbaumer‐Ochsner et al., 2012; Reite et al., 1975) but is questionable at lower altitudes (Fischer et al., 2004; Orr et al., 2018; Tseng et al., 2015). In addition, a decrease in AMS prevalence and severity over 4 days of exposure to 4300 m has been shown repeatedly by others (Beidleman et al., 2017; Fulco et al., 2011).

*Sex Differences*: Recent research suggests that women experience greater ventilatory stability at altitude and less periodic breathing than men (Caravita et al., 2015; Lombardi et al., 2013; Pramsohler et al., 2019). Even though there has been recent surge of interest in this topic, greater sleep efficiency in women compared to men at high altitude has been reported as far back as 1977 (Miller & Horvath, 1977). Sex hormones may directly impact the central medullary rhythm generator and/or modulate the peripheral chemoreflex sensitivity (Gargaglioni et al., 2019). Indirectly, sex hormones may impact cerebral blood flow and/or mechanics of lung function (Ainslie et al., 2007). Estrogen, progesterone, and testosterone are all involved in the central neural control of breathing and cyclic differences in ventilation during different phases of the menstrual phase have been repeatedly observed (Behan & Wenninger, 2008). In this study, sex differences in sleep parameters were evident across conditions (Figure 3) when all groups were combined but were not significantly different between groups. Given that there were only 3–4 women in each group, group differences may have been obscured.

One limitation of this study is that periodic breathing was not directly measured and this is the parameter that is most often reported to be increased in men compared to women even at sea level. Even though identifying sex differences in sleep quality and quantity was a secondary and not primary purpose of this study, insight can still be garnered from the fact that both DeSHr and Awak were higher at PP1 and PP2 in men compared to women while Eff was lower in men compared to women at STG1 and STG2 with all groups combined. In comparison, sSpO_2_ showed no sex differences in any condition. It has been hypothesized that increases in periodic breathing may lead to both lower and higher sSpO_2_ (Lombardi et al., 2013). The fact that sSpO_2_ did not differ between the sexes in this study, therefore, is not surprising.

The literature is controversial on whether men or women is at increased risk for developing AMS during high altitude exposure. AMS symptomatology at high altitude in men versus women has been reported to be similar (Beidleman et al., 2018), lower (Wagner et al., 2008), and higher (Murdoch, 1995) in men versus women. A recent meta‐analysis indicated that females were at higher risk for experiencing AMS (Hou et al., 2019) while a predictive model using individual data indicated that females were protected against AMS (Beidleman et al., 2013). These results, therefore, just add another data point to the controversy and support the need for further well‐controlled studies to tease out the sex differences in AMS symptomatology.

*Physical Activity Differences*: One of the benefits of this study compared to others is the controlled physical activity conditions. Under the conditions of this study, increased levels of physical activity did not impact AMS or sleep quality and quantity while staging. The debate as to whether exercise induces greater symptoms of AMS remains controversial with some reporting higher levels of AMS with exercise (Beidleman et al., 2013; DiPasquale et al., 2015; Roach et al., 2000) and others reporting no change (Mairer et al., 2013; Rupp et al., 2013; Schommer et al., 2012). Certainly, faster ascent rates during mountaineering expeditions are associated with a higher level of AMS (Bloch et al., 2009; Hackett et al., 1976; Schneider et al., 2002) and presumably, faster ascents would require greater levels of physical activity. Although not significant, there was a trend (*p* = 0.08) for the high physical activity group to experience a greater prevalence of AMS at STG1 and a reduced prevalence at PP1 with no differences at any of the other time points. This differential effect of physical activity under different exposure conditions supports the contention that physical activity may induce a greater arterial desaturation and perhaps disrupt fluid regulation (Hoyt et al., 1996) on the days of activity such that AMS tends to be greater on those days. On the other hand, desaturation during exercise may induce a greater level of acclimatization due to a larger accumulated dose of hypoxia which then reduces AMS following subsequent ascent to 4300 m.

The question of whether greater levels of physical activity impact sleep quality and quantity at altitude has only been recently explored (Aquino‐Lemos et al., 2016; Tellez et al., 2016). In the study by Tellez et al. (2016), only two bouts of moderate exercise intensity induced a lower nocturnal saturation and higher number of apnea/hypopnea events at 4000 m. On the other hand, de Aquino‐Lemos et al. (2016) reported an improvement in sleep efficiency at 4500 m altitude with two daily bouts of hypoxic exercise conducted at ~50% of maximal oxygen uptake. In our study, groups engaged in 1–2 moderate intensity hikes per day during both staging days. Therefore, the level of physical activity was similar between studies but our results do not support the finding that engaging in physical activity induces greater or lower sleep disturbances at altitude. One limitation of the previous study (Tellez et al., 2016) is a lack of baseline measurements of sleep quality and quantity which makes it difficult to determine whether the changes were due to increased physical activity or individual variability in sleep that existed prior to the intervention. Further research is warranted as altitude expeditions, competitions, and military operations typically involve an increase in physical activity.

## 5 CONCLUSION

Given the effectiveness of 2 days of staging to induce improvements in sleep quality and quantity, military personnel, mountaineers, and athletes can effectively stage at any of these altitudes prior to ascent to a higher altitude. Although all staging strategies may be equally beneficial following ascent to a higher altitude, the road to get there is not equally arduous. The 3500 and 4300 m groups experienced greater AMS and sleep disturbances while staging, while the lower altitude staging groups (2500 and 3000 m) were relatively unaffected. However, the 2500 m group was still susceptible to AMS following subsequent ascent to 4300 m indicating that acclimatization was not induced by 2 days of staging at this altitude. Therefore, 3000 m may be the optimal 2‐day staging altitude to tolerate the deleterious effects of hypoxia, achieve acclimatization, and protect against AMS during further ascent to a higher altitude.

## CONFLICT OF INTEREST

No conflict of interest for any author.

## AUTHOR CONTRIBUTIONS

JES: Data collection, data analysis, data interpretation, manuscript preparation, and final manuscript approval. SRM: Study design, data collection, data interpretation, and final manuscript approval. CSF: Study design, data collection, data interpretation, and final manuscript approval. SPA: Data collection and final manuscript approval. BAB: Study design, data collection, data analysis, manuscript preparation, and final manuscript approval.
